# Maltine in Phthisis

**Published:** 1887-03

**Authors:** William Porter

**Affiliations:** St. Louis


### *MALTINE IN PHTHISIS.*

By WILLIAM PORTER, A. M., M. D., St. Louis.

After full trial of the different oils and extract of malt preparations in both hospital and private practice, I find Maltine applicable to the greatest number of patients, and superior to any remedy of its class. Theoretically, we would expect this preparation, which has become practically officinal, to be of great value in chronic conditions of waste and malnutrition, especially as exemplified in phthisis. Being rich in *diastase, albuminoids and phosphates*, according to careful analysis, it aids in digesting farinaceous food, while in itself it is a brain, nerve and muscle producer.

In practice this hypothesis is sustained. A female patient at St. Luke's Hospital, aged thirty-five, with phthisis, signs of deposit in left upper lobe, losing flesh for six months, poor appetite and night-sweats, began taking Maltine March 13, 1880. She now weighs 121 lbs., eats well, no night-sweats, and the evidences of local disease are much less marked.

Another case of phthisis: A gentleman from Alabama, with all the physical signs of phthisis, rapidly losing health and strength. His was the remarkable gain of ten pounds *from six weeks' use of* Maltine.

These instances are sufficient for illustration, and are *duplicated many times in the experience of physicians everywhere.* There is a universal reluctance always to testify to results from medical preparations, but when, as in this case, the composition is fully known, and the profession invited to investigate the manner of preparing it, there is no reason why the remedy should not receive general approbation, provided it be worthy.—*Quarterly Epitome.*

